# Effect of short‐term consumption of yellow peas as noodles on the intestinal environment: A single‐armed pre‐post comparative pilot study

**DOI:** 10.1002/fsn3.3416

**Published:** 2023-05-10

**Authors:** Mei Yamada, Joto Yoshimoto, Tetsuya Maeda, Sho Ishii, Mikiya Kishi, Takashi Taguchi, Hidetoshi Morita

**Affiliations:** ^1^ Central Research Institute, Mizkan Holdings Co., Ltd. Handa‐Shi Japan; ^2^ New Business Development, Mizkan Holdings Co., Ltd. Tokyo Japan; ^3^ UNLOG K.K. Tokyo Japan; ^4^ Graduate School of Environmental and Life Science Okayama University Okayama Japan

**Keywords:** *Bifidobacterium longum*, fecal metabolomics, *Ruminococcus bromii*, yellow peas

## Abstract

Legumes contain dietary fiber and resistant starch, which are beneficial to the intestinal environment. Here, we investigated the effects of yellow pea noodle consumption on the gut microbiota and fecal metabolome of healthy individuals. This single‐armed pre‐post comparative pilot study evaluated eight healthy female participants who consumed yellow pea noodles for 4 weeks. The gut microbiota composition and fecal metabolomic profile of each participant were evaluated before (2 weeks), during (4 weeks), and after (4 weeks) daily yellow pea noodle consumption. 16S rRNA gene sequencing was performed on stool samples, followed by clustering of operational taxonomic units using the Cluster Database at High Identity with Tolerance and integrated QIIME pipeline to elucidate the gut microbiota composition. The fecal metabolites were analyzed using capillary electrophoresis time‐of‐flight mass spectrometry and liquid chromatography time‐of‐flight mass spectrometry. Compared to day 0, the relative abundances of five bacterial genera (*Bacteroides, Bilophila, Hungatella, Parabacteroides*, and *Streptococcus*) in the intestinal microbiota significantly decreased, wherein those of *Bifidobacterium longum* and *Ruminococcus bromii* were increased on day 29 and decreased to the basal level (day 0) on day 57. Fecal metabolomic analysis identified 11 compounds showing significant fluctuations in participants on day 29 compared to day 0. Although the average levels of short‐chain fatty acids in participants did not differ significantly on day 29 compared to those on day 0, the levels tended to increase in individual participants with >8% relative abundance of *R. bromii* in their gut microbiota. In conclusion, incorporating yellow peas as a daily staple may confer human health benefits by favorably altering the intestinal environment.

## Graphical abstract

Short‐term consumption of yellow pea noodles for 4 weeks increased the relative abundances of Bifidobacterium longum and Ruminococcus bromii in the gut microbiota of healthy participants. Consumption of yellow pea noodles for 4 weeks did not alter the fecal metabolomic profile in overall data, but the intestinal microbiota compositions varied at the individual level. Consuming yellow peas as a daily dietary staple may confer human health benefits by altering the gut microbiota composition to favor beneficial microflora.

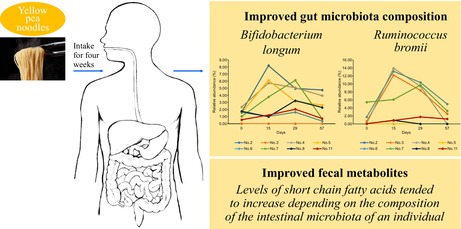

## 1 INTRODUCTION

The human intestinal tract is a complex consortium of over 300 bacterial species and trillions of individual bacteria, which comprise the intestinal microbiota and form a unique ecosystem adapted to the intestinal environment (Quigley, 2013; Sender et al., 2016). The gut microbiome modulates gastrointestinal development, confers enhanced metabolic capabilities, and protects against pathogens (Bäckhed et al., 2005). Several studies have revealed the crucial role of diet in determining the composition of the human gut microbiota. Strategic dietary interventions have been shown to modulate the abundance of specific species and have gained interest in the development of novel therapeutic methods for disease control and prevention (David et al., 2014; Kolodziejczyk et al., 2019; Walker et al., 2011).

Recent studies have clarified that intestinal bacterial metabolites, such as organic acids, short‐chain fatty acids (SCFAs), and branched amino acids, contribute to health maintenance. SCFAs produced by intestinal bacteria impart various health effects, such as serving as a source of energy for intestinal epithelial cells, lowering intestinal pH, suppressing the growth of harmful bacteria, preventing obesity, reducing intestinal inflammation, and regulating immune functions (Ríos‐Covián et al., 2016). Species and occupancy rates of intestinal bacteria, as well as diet composition, significantly affect the production of these metabolites (Green et al., 2016; Li et al., 2018). In particular, dietary fiber and resistant starch (RS) are important components that contribute to maintaining the intestinal environment and normal defecation (Bird et al., 2010).

Legumes, rich in proteins, minerals, and phytochemicals such as polyphenols, are recognized as highly nutritious foods (Marinangeli et al., 2020). Consumption of legumes leads to various beneficial effects, such as sustained satiety, body weight improvement, metabolic syndrome prevention, and extension of healthy life expectancy (Darmadi‐Blackberry et al., 2004; Polak et al., 2015). Legumes are also high in dietary fiber and RS, which are beneficial for reducing postprandial blood glucose levels (Clemente & Olias, 2017; Mayengbam et al., 2019). Several clinical trials have explored the favorable role of legume consumption in the modulation of the human gut microbiota and metabolite profiles (Baxter et al., 2018; Fernando et al., 2010; Finley et al., 2007; Kadyan et al., 2022; Sheflin et al., 2017). For example, the consumption of canned chickpeas (200 g/day) for 3 weeks reduced the abundance of pathogenic and putrefactive gut bacterial species in healthy adults (Fernando et al., 2010). Similarly, consuming cooked navy beans at 35 g/day for 28 days increased gut bacterial diversity and altered gut microbial composition compared to the baseline in colorectal cancer survivors (Sheflin et al., 2017). Another study showed that navy beans significantly altered the stool metabolome and metabolic pathways involved in maintaining colon health in cancer survivors (Baxter et al., 2018). Owing to their health and environmental benefits, legumes are increasingly consumed not only as unprocessed foods but also as processed foods such as noodles, plant‐based meat, and milk alternatives. Despite these benefits, the actual intake of legumes is lower than optimal (~60 g) in many regions worldwide. A diet low in legumes increases the risk of heart disease, and this risk is higher in younger age groups than in older age groups (GBD 2017 Diet Collaborators, 2019). In Japan, the intake of legumes, a rich source of dietary fiber, is low among younger people. The dietary fiber intake is low, particularly in females aged 20–50 years (<16 g/day vs. ≥24 g/day [recommended limit]; Committee for Development of the “Dietary Reference Intakes for Japanese” & Ministry of Health, Labour and Welfare, Japan, 2020; Ministry of Health, Labour, and Welfare, Japan, 2020).

Dietary fiber and RS are fermented by intestinal bacteria and exert favorable effects on the intestinal environment (Baxter et al., 2018; Mayengbam et al., 2019; Sheflin et al., 2017), which is dependent on the structure of dietary fiber and RS in food. For example, SCFA production during in vitro fecal fermentation with pinto bean cells as a substrate increased after enzymatic treatment of beans compared to intact beans, indicating the effects of cell wall integrity of pinto beans on the modulation of the microbiota composition (Guan et al., 2020). However, it remains unclear whether processed legumes can consistently provide the health benefits of unprocessed legumes. Moreover, few studies have explored the effects of legume consumption as a staple food source on the intestinal environment. In addition, inculcating the habit of eating legumes in people who have not been consuming them daily can be challenging. Like other legumes, yellow peas are enriched with protein and dietary fiber, thus attracting attention as a plant‐based meat alternative (Ferawati et al., 2021; Smith et al., 2012). We previously reported that yellow pea noodles are palatable and showed the possibility of their continuous consumption as a staple food (Yoshimoto et al., 2020). However, knowledge of the effects of continuous consumption of such foods on the gut microbiota is limited.

Therefore, the present pilot study aimed to investigate the effects of consuming yellow pea noodles continuously for 28 days on the gut microbiota composition and fecal metabolomic profiles of healthy individuals.

## 2 MATERIALS AND METHODS

### 2.1 Participants

**FIGURE 1:**
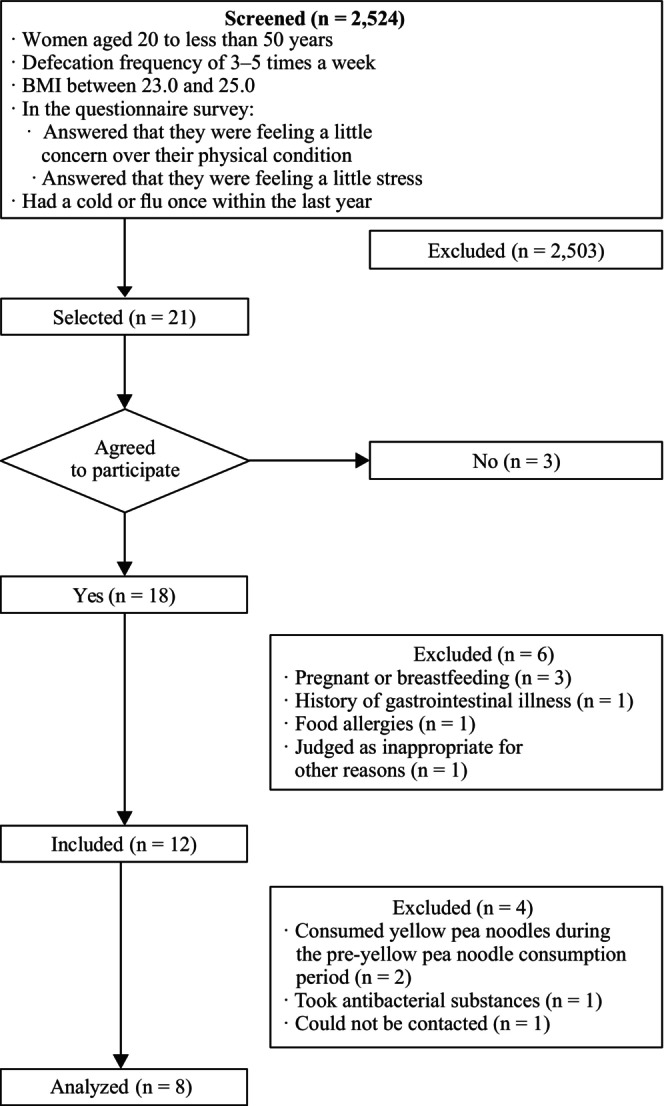
Flowchart of screening and recruitment of participants for the study.

The participants in this study were recruited through an advertisement sent to users of the UNLOG app in Japan. A total of 2524 individuals were recruited after responding to the advertisement. Individuals were screened based on the following inclusion criteria: (1) women aged 20 to less than 50 years, (2) defecation frequency 3–5 times per week, (3) body mass index (BMI) between 23.0 and 25.0, (4) answered that they were feeling a little concern over their physical condition, (5) answered that they were feeling a little stress, and (6) experienced a cold or flu once within the last year. A total of 21 candidates fulfilled the above criteria, among whom 18 agreed to participate. Ten participants were excluded after deviating from the study conditions, leaving eight participants in the study (Figure 1).

### 2.2 Test material

Yellow pea noodles made exclusively from unshelled yellow pea flour were obtained from ZENB JAPAN Co., Ltd. (Aichi, Japan). The prepared boiled yellow pea noodles (100 g) contained 65 g water, 7.1 g protein, 26.2 g carbohydrates, 7.4 g total dietary fiber, and 2.1 g RS.

### 2.3 Study schedule

**FIGURE 2:**
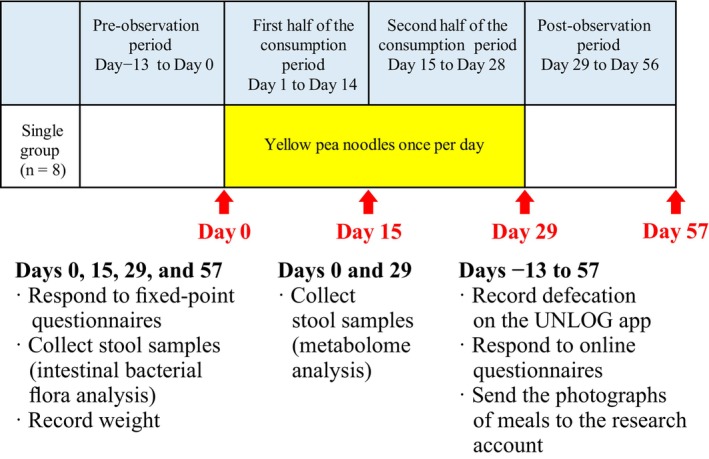
Study schedule for 10 weeks, including 2‐week pre‐observation period (days −13 to 0), 4 weeks of yellow pea noodle consumption period (days 1 to 28), and 2‐week post‐observation period (days 29 to 56). The start of yellow pea noodle consumption was day 1.

This study employed a single‐arm pre‐post comparison strategy. The total study period was 10 weeks (Figure 2), comprising a pre‐observation period of 2 weeks, an experimental period of 4 weeks, and a post‐observation period of 4 weeks. During the experimental period, the participants consumed boiled yellow pea noodles (200 g per serving) once daily, with a seasoning of their choice. Participants were not allowed to consume yellow pea noodles during the pre‐ and post‐observation periods. Additionally, participants were not allowed to consume new medications, supplements, or foods that could affect the intestinal microbiota during the entire study period, such as probiotics, food products fortified with oligosaccharides or dietary fiber, or health foods that improve constipation. However, participants already taking such substances were allowed to continue taking them during the entire study period, maintaining a consistent intake quantity.

During the total study period, participants recorded their defecation schedules and responded to online questionnaires using the UNLOG app. DialBetics, a smartphone‐based application, was used to record the participants' meals (Waki et al., 2014). The participants were instructed to take photographs of all meals and send them to the research account. Subsequently, meal images were analyzed using DialBetics to quantify the energy and nutrient intake. Stool samples were collected on days 0, 15, 29, and 57 for further analysis. The study protocol was registered in the University Hospital Medical Information Network Clinical Trials Registry (UMIN‐CTR; UMIN000041442).

### 2.4 Metabolite extraction and analysis

#### 2.4.1 Metabolite extraction for capillary electrophoresis–mass spectrometry

Approximately 30–50 mg fecal sample (collected on days 0 and 29) was mixed with 500 μL Milli‐Q water containing internal standards (H3304‐1002; Human Metabolome Technologies, Inc. [HMT], Tsuruoka, Japan). The mixture was centrifuged at 2300× *g* for 5 min at 4°C, after which 350 μL supernatant was centrifugally filtered through a 5‐kDa cutoff filter (UltrafreeMC‐PLHCC, HMT) at 9100× *g* for 120 min at 4°C to remove macromolecules. Subsequently, the filtrate was evaporated to dryness under vacuum and reconstituted in 20 μL Milli‐Q water. Metabolomic analysis was performed at HMT.

#### 2.4.2 Metabolite extraction for liquid chromatography‐mass spectrometry

Approximately 30–50 mg fecal sample was mixed with 1000 μL methanol containing internal standards (H3304‐1002, HMT). The mixture was centrifuged at 2300× *g* for 5 min at 4°C, after which 300 μL supernatant was centrifugally filtered through a 3‐kDa cutoff filter (Nanosep 3 K Omega; Pall Corporation, Port Washington, NY, USA) at 9100× *g* for 30 min at 4°C to remove macromolecules. Subsequently, the filtrate was evaporated to dryness under nitrogen and reconstituted in 400 μL 50% isopropanol (v/v). Metabolomic analysis was performed at HMT.

#### 2.4.3 Metabolomic analysis (dual scan)

Metabolomic analysis was conducted according to HMT's Dual Scan package, using capillary electrophoresis time‐of‐flight mass spectrometry (CE‐TOF‐MS) and liquid chromatography time‐of‐flight mass spectrometry (LC‐TOF‐MS) following the methods described previously (Ohashi et al., 2008; Ooga et al., 2011). Briefly, CE‐TOF‐MS analysis was performed using an Agilent CE system equipped with an Agilent 6210 TOF mass spectrometer (Agilent Technologies, Inc., Santa Clara, CA, USA). LC‐TOF‐MS analysis was performed using an Agilent 1200 high‐performance liquid chromatography system fitted with an Agilent 6210 TOF mass spectrometer (Agilent Technologies). The systems were controlled using Agilent G2201AA ChemStation software version B.03.01 for CE‐TOF‐MS (Agilent Technologies) and MassHunter for LC‐TOF‐MS (Agilent Technologies). The scan range was *m/z* 50–1000. Peaks were extracted using MasterHands, automatic integration software (Keio University, Tsuruoka, Yamagata, Japan) to obtain peak information, including *m/z*, peak area, migration time (MT) for CE‐TOFMS, and retention time (RT) for LC‐TOFMS analyses (Sugimoto et al., 2010). Signal peaks corresponding to isotopomers, adduct ions, and other product ions of known metabolites were excluded. The remaining peaks were annotated according to the HMT metabolite database, based on their *m*/*z* values and MTs or RTs. Then, areas of the annotated peaks were normalized to internal standards and sample amount to obtain relative levels of each metabolite. A total of 110 primary metabolites were quantified based on one‐point calibrations using their respective standard compounds.

#### 2.4.4 Acetic acid quantification

Acetic acid was quantified using the Acetate Colorimetric Assay Kit (Sigma‐Aldrich, St. Louis, MO, USA). Briefly, a 30–50 mg fecal sample was mixed with 500 μL Milli‐Q water containing internal standards (H3304‐1002, HMT). The mixture was centrifuged at 2300× *g* for 5 min at 4°C, after which 350 μL supernatant was centrifugally filtered using a 5‐kDa cutoff filter (Ultrafree‐MC PLHCC, HMT) at 9100× *g* for 120 min at 4°C to remove macromolecules. Subsequently, 15 μL filtrate was mixed with the assay buffer supplied with the kit. The absorbance of the sample was measured at 450 nm using an Infinite 200 PRO microplate reader (Tecan, Männedorf, Switzerland). Five‐point calibration curves were prepared using standard compounds to estimate acetate concentrations in the fecal samples.

### 2.5 Sample collection and DNA extraction

Rice grain‐sized fecal samples were collected on days 0, 15, 29, and 57, using a fecal collection kit (Techno Suruga Laboratory, Shizuoka, Japan). After vigorous mixing, the samples were stored at room temperature for 7 days until DNA extraction. Genomic DNA was isolated using a NucleoSpin Microbial DNA Kit (Macherey‐Nagel, Düren, Germany), following the manufacturer's instructions. Briefly, a 500 μL fecal sample was placed in a microcentrifuge tube containing 100 μL elution buffer provided with the kit. The mixture was then placed in a NucleoSpin bead tube containing proteinase and homogenized at 30 Hz for 12 min using a TissueLyser LT sample disrupter (Qiagen, Hilden, Germany). The subsequent extraction procedure was performed according to the manufacturer's instructions. DNA samples were purified using the AMPure XP system (Beckman Coulter Inc., Brea, CA, USA).

### 2.6 Sequencing of 16S rRNA gene

A two‐step polymerase chain reaction (PCR) was performed on the purified DNA samples to obtain the sequencing libraries. In the first step, the 16S rRNA (V3–V4 region) was amplified using the primer pairs 341F (5′‐TCGTCGGCAGCGTCAGATGTGTATAAGAGACAGCCTACGGGNGGCWGCAG‐3′) and 806R (5′‐GTCTCGTGGGCTCGGAGATGTGTATAAGAGACAGGGACTACHVGGGTWTCTAAT′), and the PCR product was purified using the AMPure XP system (Beckman Coulter Inc.). In the second step, PCR was performed on the first PCR product using the 16S rRNA amplicon as a template and a primer with an index sequence unique to each sample. The PCR product was purified using the AMPure XP system (Beckman Coulter Inc.) and used to construct the sequencing library. The double‐stranded DNA was quantified using a Qubit Fluorometer (Thermo Fisher Scientific, Waltham, MA, USA), and the indexed samples were mixed and sequenced using the MiSeq Reagent Kit v3 (Illumina Inc., San Diego, CA, USA) at Takara Bio's Biomedical Center (Takara Bio, Shiga, Japan).

### 2.7 Data analysis

Taxonomic identification was performed by clustering operational taxonomic units (OTUs) using the Cluster Database at High Identity with Tolerance (CD‐HIT‐OTUs) and integrated QIIME pipeline for 16S rRNA (Caporaso et al., 2010; Li et al., 2012). Briefly, sequence reads 1 and 2 were assembled and clustered using CD‐HIT‐OTUs, and representative sequences were extracted. The QIIME pipeline was used to assign phylogenetic nomenclature to the representative sequences.

### 2.8 Statistical analysis

Statistical analysis of the metabolomic data was conducted using Welch's *t*‐test. Paired *t*‐tests were used to compare other data using the bell curve in Excel (Social Survey Research Information Co., Ltd., Tokyo, Japan). *p*‐Values <.05 were considered to be statistically significant.

## 3 RESULTS

### 3.1 Effect of yellow pea noodle consumption on body weight and defecation frequency

All participants consumed at least 26 servings of boiled yellow pea noodles (200 g/day/serving). The average body weights of participants did not differ significantly (*p* > .05) before (day 0, 58.9 ± 3.4 kg) and after daily consumption (day 29, 58.9 ± 4.0 kg) of yellow pea noodles. Additionally, the average body mass index (BMI) values of participants on day 0 (23.5 ± 0.7) and day 29 (23.5 ± 0.8) and the number of defecations between days −13 to 0 (10.5 ± 2.9) and days 15 to 28 (11.0 ± 2.9) did not differ significantly (*p* > .05; Table S1).

### 3.2 Effect of yellow pea noodle consumption on intestinal metabolites

Fecal metabolomic analysis identified 11 compounds that displayed significant fluctuations in participants on day 29 compared to day 0 (Table S2). As shown in Table S3, the average acetic acid levels in the feces of participants did not differ significantly (*p* > .05) between day 0 (38.26 ± 13.92 μmol/g) and day 29 (41.00 ± 21.63 μmol/g). Similarly, propionic acid and butyric acid levels in feces did not differ significantly on day 29 compared to those on day 0 (Tables S4 and S5).

### 3.3 Effect of yellow pea noodle consumption on intestinal microbiota

The relative abundances (percentage of reads) of five bacterial genera (*Bacteroides, Bilophila, Hungatella, Parabacteroides*, and *Streptococcus*) in the intestinal microbiota of participants significantly decreased on day 29 compared to those on day 0 (Table 1).

**TABLE 1:** Significantly fluctuating genera during yellow pea noodle consumption.

Genus	Relative abundance(%)		Ratio (day 29/day 0)	*p*‐Value*
	Mean ± standard deviation (SD)			
	Day 0	Day 29		
*Bacteroides*	19.35 ± 12.12	14.10 ± 10.96	0.73	.048
*Bilophila*	0.10 ± 0.10	0.04 ± 0.05	0.37	.045
*Hungatella*	0.04 ± 0.03	0.02 ± 0.02	0.50	.031
*Parabacteroides*	2.16 ± 1.56	1.07 ± 0.77	0.50	.044
*Streptococcus*	0.98 ± 0.75	0.43 ± 0.37	0.44	.010

*Note*: All values represent the mean ± standard deviation (SD) of eight participants.

* Significant difference between days 0 and 29; *p* < .05 (paired *t*‐test).

**FIGURE 3:**
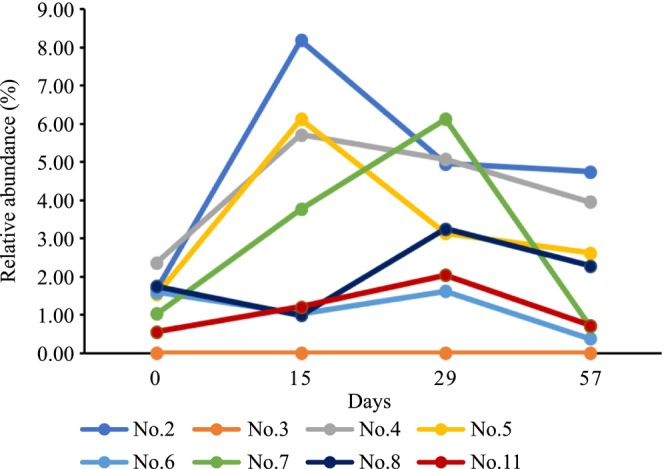
Fluctuations in the relative abundance of *Bifidobacterium longum* (OTU6) among participants.

**FIGURE 4:**
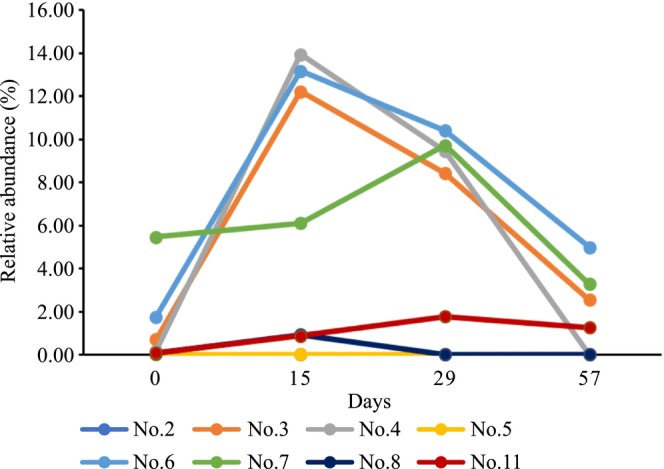
Fluctuations in the relative abundance of *Ruminococcus bromii* (OTU8) among participants.

The average relative abundances of eight OTUs were significantly changed in all participants on day 29 compared to those on day 0. In particular, the relative abundances of OTU6 (*Bifidobacterium longum*) and OTU8 (*Ruminococcus bromii*) increased significantly on day 29 compared with those on day 0. The relative abundances of the remaining six OTUs decreased significantly on day 29 compared with those on day 0 (Table 2). Analyzing individual differences in intestinal microbiota composition revealed that in seven participants (no. 2, 4, 5, 6, 7, 8, and 11), the relative abundance of OTU6 (*Bifidobacterium longum*) increased clearly on day 29 compared to that on day 0, but was reduced to the basal level on day 57. However, this bacterium was not detected in participant no. 3 during the entire experimental period, likely because levels were below the detection limit (Figure 3). Similarly, OTU8 (*R. bromii*) was detected in six participants (nos. 3, 4, 6, 7, 8, and 11) on day 0. The relative abundance of this bacteria increased clearly among the six participants on days 15 and 29 compared with that on day 0. Similar to OTU6 (*Bifidobacterium longum*), the relative abundance of OTU8 (*R. bromii)* decreased to the basal level in these participants on day 57. OTU8 (*R. bromii)* was not detected in participant nos. 2 and 5, likely because levels were below the detection limit (Figure 4).

**TABLE 2:** Significantly fluctuating operational taxonomic units during yellow pea noodle consumption.

OTU	Taxonomy		Relative abundance (%)		Ratio (day 29/ day 0)	*p*‐Value*
			Mean ± SD			
			Day 0	Day 29		
OTU6	*Bifidobacterium*	*Bifidobacterium longum*	1.31 ± 0.75	3.27 ± 2.04	2.49	.014
OTU8	*Ruminococcus*	*Ruminococcus bromii*	1.03 ± 1.89	4.97 ± 4.90	4.83	.030
OTU48	*Dorea*	*Dorea longicatena*	0.43 ± 0.43	0.18 ± 0.23	0.41	.045
OTU68	*Streptococcus*	*Streptococcus* sp._s207	0.93 ± 0.71	0.41 ± 0.36	0.44	.012
OTU77	*Parabacteroides*	*Bacterium* nlae‐zl‐p344	1.12 ± 1.01	0.54 ± 0.70	0.48	.044
OTU133	*Bilophila*	*Bacterium* nlae‐zl‐h528	0.10 ± 0.10	0.04 ± 0.05	0.37	.045
OTU134	*Hungatella*	*Bacterium* nlae‐zl‐h504	0.04 ± 0.03	0.02 ± 0.02	0.50	.031
OTU167	*Clostridium_xlvb*	*Clostridiales* bacterium 21‐4c	0.19 ± 0.21	0.03 ± 0.05	0.15	.045

*Note*: All values are the mean ± standard deviation (SD) of eight participants; Day 0, before consumption; Day 29, after consumption of yellow pea noodles.

Abbreviation: OTU, operational taxonomic unit.

* Significant difference between days 0 and 29; *p* < .05 (Paired *t*‐test).

## 4 DISCUSSION

**FIGURE 5:**
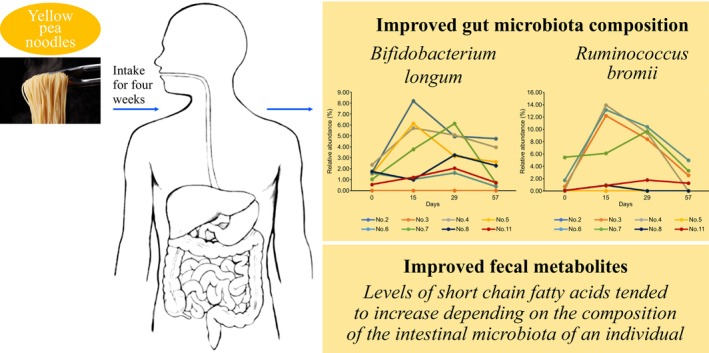
Effects of yellow pea noodle consumption on gut microbiota and fecal metabolomic profile of healthy adults. Yellow pea noodle consumption did not induce major changes in the metabolomic profile in overall data but favorably altered individual gut microbiota compositions, suggesting that long‐term intake of yellow pea noodles may confer health‐promoting effects.

The present study evaluated the effects of yellow pea noodle consumption on the gut microbiota and fecal metabolome of healthy adults. The study findings suggest that the consumption of yellow pea noodles clearly and favorably altered individual compositions of the gut microbiota (Figure 5).

Yellow pea noodle consumption did not significantly affect the average body weight, BMI, or defecation frequency of participants. Several studies have reported the beneficial effects of peas or high‐fiber‐containing foods on weight gain (Abete et al., 2009; Ito et al., 2023; Jarrar et al., 2019; Lambert et al., 2017). No fluctuations in body weight were observed in a trial in which participants with BMIs between 18.5 and 30 consumed a high‐fiber cereal (Jarrar et al., 2019). Furthermore, the potential of yellow pea fiber to induce weight loss in obese individuals has been previously reported (Lambert et al., 2017). Taken together, these studies suggest that fiber‐rich yellow pea noodles could provide a food commodity to help reduce body weight fluctuations in individuals with high BMIs. Nevertheless, the average BMI of participants in the current study remained stable at 23.5, indicating that all participants maintained their healthy body weight throughout the study period. Further studies are required to explore the benefits of yellow pea noodle consumption in obese individuals.

The intestinal microbiota analysis in the present study revealed that the relative abundances of *Bifidobacterium longum* and *R. bromii* significantly increased on day 29 compared to those on day 0, which suggested the benefits of yellow pea noodle consumption. However, these levels returned to pre‐consumption levels when yellow pea noodle consumption was stopped, suggesting that the relative abundances of these bacteria were reversible and caused by the continuous consumption of yellow pea noodles. *Bifidobacterium longum* ferments dietary fiber (Iwata et al., 2009). *R. bromii* quickly degrades RS and is particularly important for the fermentation of aged starch with a native granular structure (RS2) and retrograded starch after heating and cooling (RS3) (Ze et al., 2012). Boiled yellow pea noodles are rich in total dietary fiber (7.4%) and RS (2.1%). The meal records revealed a significant increase in the average daily intake of dietary fiber in participants during the yellow pea noodle consumption period (20.03 g) compared to that during the pre‐observation (10.42 g) and post‐observation (9.40 g) periods. However, energy intake and other major nutrients (protein, fat, and salt equivalents) did not change significantly. Therefore, we speculate that the dietary fiber from yellow pea noodles contributed to the increased relative abundance of these bacteria during the consumption period. Notably, bifidobacteria are predominant in the Japanese intestinal flora. In particular, *Bifidobacterium longum* is the only bifidobacterium reportedly present in both infants and adults over long periods. An estimated 87% of the total Japanese population harbors this bacterium (Odamaki et al., 2018). Furthermore, the gut enterotype of the Japanese population is classified as *Ruminococcus* type, with a high relative abundance of *Ruminococcus* bacteria (Arumugam et al., 2011). Therefore, the favorable effects of yellow pea noodle consumption on the intestinal microbiota could be adaptable for Japanese.

Acetic acid is a major metabolite of *Bifidobacterium longum*, whereas acetic acid, butyric acid, and propionic acid are the major metabolites of *R. bromii* (Bianchi et al., 2019). However, we found no significant changes in these metabolites (Tables S2–S5). We found an increased abundance of *Bifidobacterium longum* (Table 2), whereas that of *Bacteroides* and *Parabacteroides*, whose major metabolite is acetic acid, decreased during yellow pea noodle consumption (Table 1). These results suggest that changes in acetic acid may have been counterpoised by alterations in the microbial composition. On the contrary, participants with ≥8% relative abundance of *R. bromii* in the gut microbiota on day 29 (nos. 3, 4, 6, and 7) tended to produce higher levels of SCFAs compared to other participants (Tables S3–S5). This suggests that the effects of yellow pea noodles on metabolites may vary depending on the composition of the intestinal microbiota of an individual. These findings align with previous reports in which RS fermentation was enhanced in individuals harboring *R. bromii*, but not in those with undetectable *R. bromii* levels (Louis et al., 2007; Walker et al., 2011). Moreover, this may explain the nonincrease in the relative abundances of *Bifidobacterium longum* and *R. bromii* on day 29 in individuals with undetectable levels of these bacteria on day 0.

*Bifidobacterium longum* is a probiotic that benefits the intestinal environment and suppresses inflammation (Yao et al., 2021). *R. bromii* is a keystone species that degrades RS and favorably alters the composition of the gut microbiota (Scott et al., 2015). Moreover, several studies have reported the health benefits of SCFAs produced by the intestinal microbiota (Rauf et al., 2022). For example, acetic acid produced by *Bifidobacterium longum* protects against enteropathogenic infections (Fukuda et al., 2011). Taken together, the study findings suggest that consuming yellow peas as a daily staple may confer human health benefits.

Consumption of yellow pea noodles caused significant changes in the average relative abundances of five bacterial genera (*Bacteroides, Bilophila, Hungatella, Parabacteroides*, and *Streptococcus*) in the intestinal microbiota. Of these, the average relative abundance of *Bilophila* decreased below 50% on day 29 compared to day 0 (Table 1). *Bilophila wadsworthia* is the only species reported in *Bilophila*, and the abundance of this species was shown to be increased by an animal‐based diet (Odamaki et al., 2016). Studies have indicated that it destroys the intestinal mucosa to produce hydrogen sulfide, a toxic substance, and is involved in the induction of inflammation in the intestinal tract (David et al., 2014; Devkota et al., 2012). *Bilophila wadsworthia* comprises approximately <0.01%–0.2% of normal human intestinal microbiota (Baron, 1997; Dostal Webster et al., 2019; Vandeputte et al., 2017). In the present study, the average relative abundance of *Bilophila* was less than 0.1% on day 29. This finding indicates that dietary habits of consuming yellow pea noodles as a staple food promote not only the intake of RS and dietary fiber in yellow peas but also reduce health risks derived from animal‐based diets and improve the intestinal environment. Furthermore, Odamaki et al. (2016) showed that the animal‐diet‐induced increase in the abundance of *Bilophila* was restored by the intake of yogurt supplemented with *Bifidobacterium longum*, which could be attributed to the bile salt hydrolase activity of bifidobacteria (Begley et al., 2006). Our study showed that the yellow pea noodles consumption resulted in a reduction in *Bilophila* despite no restrictions on the intake of animal‐based diets. Therefore, we inferred that the abundance of bifidobacteria increased by fermentation of RS and dietary fiber in yellow pea noodles could have reduced the growth of bile‐using bacteria, *Bilophila*. Moreover, the dietary habit of eating yellow pea noodles could have made participants feel full, leading to reduced consumption of animal‐based diets and, therefore, a reduced abundance of *Bilophila*. Taken together, these results suggest that dietary habits with a high degree of freedom to consume yellow pea noodles once daily with any seasoning at any time and without restrictions on intake of animal‐based diets contribute to reducing long‐term health risks and can be continued with little patience.

This study revealed a significant decrease in the relative abundance of five bacterial genera (Table 1) and significant changes in 11 metabolites in the feces (Table S2) during the yellow pea noodle consumption period. We showed that lysophosphatidylcholine (LPC; 12:0) and the genera *Bilophila* and *Bacteroides* in the feces were significantly reduced during yellow pea noodle consumption. These findings are consistent with those of previous animal studies (Tang et al., 2021; Yang et al., 2022). To our knowledge, this is the first study to reveal a new correlation between the human intestinal microbiota and the fecal metabolome. It has been suggested that LPC abundance promotes the release of inflammatory cytokines and is associated with intestinal inflammation (Tagesson et al., 1985; Tang et al., 2021) and epithelial barrier function. In vitro and in vivo assessments have shown that LPC treatment damages the tight junctions of colonic epithelial cells. Therefore, we speculate that the consumption of yellow pea noodles reduces LPC production and suppresses inflammation and epithelial barrier damage in the human intestine. However, these findings need to be validated in future studies. Furthermore, because the molecular structures of the LPCs detected in previous studies and in this study could be different, further studies are necessary to confirm the effects of yellow pea noodles on LPC abundance.

The present study had several limitations. First, the study was only conducted with Japanese participants. Gut enterotypes are known to vary by race and ethnicity; hence, the findings may not be generalizable to other populations. Second, the study duration was short. As the effects of yellow pea noodles on the intestinal microbiota composition were reversed upon cessation of consumption, evaluating its effects should be conducted over a longer duration. Third, the results of this study indicated that *Bifidobacterium longum* and *R. bromii* degrade the dietary fiber and RS present in yellow pea noodles; however, the mechanism of decomposition remains unknown. Further in vitro studies are required to clarify how these bacteria degrade the dietary fiber and RS in yellow peas. Fourth, this study was limited to evaluating changes in the intestinal microbiota and associated metabolites. Evaluating the effects of yellow pea noodle consumption on other factors, such as obesity and inflammatory markers, could further strengthen its applicability as a daily staple.

In conclusion, this pilot study revealed the favorable effects of yellow pea noodle consumption on the composition of the gut microbiota, providing evidence that yellow pea noodle consumption is beneficial for human health. Furthermore, this study used an app that recorded the participants' diets, including yellow pea noodles, and their physical conditions. The benefit of such apps in helping participants become aware of healthy dietary lifestyles and providing motivation for long‐term continuation should be evaluated.

## AUTHOR CONTRIBUTIONS

**Mei Yamada:** Formal analysis (lead); visualization (lead); writing – original draft (lead); writing – review and editing (lead). **Joto Yoshimoto:** Formal analysis (supporting); methodology (lead); visualization (supporting); writing – original draft (supporting). **Tetsuya Maeda:** Formal analysis (supporting); methodology (supporting). **Sho Ishii:** Conceptualization (supporting); writing – review and editing (supporting). **Mikiya Kishi:** Conceptualization (lead); writing – review and editing (supporting). **Takashi Taguchi:** Methodology (supporting); supervision (supporting). **Hidetoshi Morita:** Supervision (lead); writing – review and editing (supporting).

## CONFLICT OF INTEREST STATEMENT

This research was funded by Mizkan Holdings Co. Ltd. M.Y., J.Y., T.M., S.I., and M.K. are employees of Mizkan Holdings Co., Ltd. None of the principal investigators involved in this study or their family members is a shareholder of Mizkan Holdings Co., Ltd., or company officers, directors, or advisors. T.T. and H.M. declare that they have no conflict of interest.

## ETHICS STATEMENT

This study protocol was approved by the ethics committees of Mizkan Holdings Co., Ltd., Aichi, Japan, and UNLOG K.K, Tokyo, Japan.

## INFORMED CONSENT

The participants provided informed consent prior to the study in accordance with the Declaration of Helsinki.

## Supporting information


Table S1‐S5.

## Data Availability

The nucleotide sequence data obtained in this study are available in the DDBJ Sequence Read Archive under accession numbers DRR353683–DRR353714. Data supporting the findings of this study are available from the corresponding author upon reasonable request.
