# Supplementary material for: Effect of short‐term consumption of yellow peas as noodles on the intestinal environment: A single‐armed pre‐post comparative pilot study
Source: Food Sci Nutr. 2023 May 10;11(8):4572–82. doi: 10.1002/fsn3.3416 (PMC10420782; doi:10.1002/fsn3.3416)
Supplement: Supplementary file 1 — Table S1‐S5. [file FSN3-11-4572-s001.docx]

**SUPPLEMENTARY MATERIALS**

**Table S1.** Body weight fluctuations and defecation frequency of participants during the study.

| Participant No. | Sex | Age  (years) | Height (cm) | Body weight | | BMI | | Number of defecations | |
| --- | --- | --- | --- | --- | --- | --- | --- | --- | --- |
|  |  |  |  | Day  0 | Day 29 | Day 0 | Day 29 | ^†^Days −13 to 0 | Days 15 to 28 |
| 2 | Female | 39 | 152.0 | 55.9 | 53.0 | 24.2 | 22.9 | 13 | 14 |
| 3 | Female | 23 | 153.0 | 56.4 | 56.6 | 24.1 | 24.2 | 12 | 9 |
| 4 | Female | 27 | 158.0 | 56.4 | 56.0 | 22.6 | 22.4 | 11 | 7 |
| 5 | Female | 28 | 155.0 | 55.5 | 56.1 | 23.1 | 23.4 | 8 | 13 |
| 6 | Female | 39 | 160.0 | 59.9 | 61.2 | 23.4 | 23.9 | 7 | 12 |
| 7 | Female | 40 | 158.0 | 61.0 | 62.0 | 24.4 | 24.8 | 11 | 13 |
| 8 | Female | 40 | 162.0 | 60.4 | 61.0 | 23.0 | 23.2 | 7 | 7 |
| 11 | Female | 22 | 168.0 | 65.3 | 64.9 | 23.1 | 23.0 | 15 | 13 |
| Mean |  | 32.3 | 158.3 | 58.9 | 58.9 | 23.5 | 23.5 | 10.5 | 11.0 |
| SD |  | 8.0 | 5.2 | 3.4 | 4.0 | 0.7 | 0.8 | 2.9 | 2.9 |

Day 0, before consumption; day 29, after consumption of yellow pea noodles; ^†^pre-observation period, days −13 to 0; day 1 is the start of yellow pea noodle consumption; BMI, body mean index; SD, standard deviation.

**Table S2.** Fecal metabolites that fluctuated significantly during yellow pea noodle consumption.

| HMT DB | *m/z* | MT/RT | Relative area | | | | Comparative analysis | |  |
| --- | --- | --- | --- | --- | --- | --- | --- | --- | --- |
| Compound name |  |  | Day 0 | | Day 29 | |  | |  |
|  |  |  | Mean | SD | Mean | SD | Ratio (Day 29/Day 0) | P-value* | |
| Xanthurenic acid | 204.03 | 10.74 | 1.6E-05 | 4.7E-06 | 3.2E-05 | 2.7E-06 | 1.98 | 0.012 | |
| 7-Dehydrocholesterol-1 Desmosterol-1 | 367.33 | 15.86 | 1.2E-04 | 7.0E-05 | 2.4E-04 | 7.1E-05 | 2.03 | 0.019 | |
| 19-Hydroxyandrostenedione | 303.20 | 8.31 | 3.1E-04 | 3.8E-05 | 7.4E-05 | 3.3E-05 | 0.24 | 0.020 | |
| Glycerol | 93.06 | 18.35 | 1.4E-02 | 2.0E-03 | 1.1E-02 | 2.6E-03 | 0.79 | 0.022 | |
| Lysophosphatidylethanolamine (18:1)-2 | 478.30 | 13.43 | 3.5E-05 | 1.2E-05 | 2.1E-05 | 7.3E-06 | 0.60 | 0.022 | |
| Lysophosphatidylcholine (12:0) | 440.29 | 11.79 | 1.0E-04 | 5.4E-05 | 4.2E-05 | 4.5E-06 | 0.42 | 0.029 | |
| Oxypurinol | 151.03 | 6.45 | 1.4E-04 | 4.8E-05 | 8.8E-05 | 3.1E-05 | 0.62 | 0.030 | |
| Prostaglandin F2α | 353.23 | 6.13 | 3.3E-05 | 8.0E-06 | 1.9E-05 | 1.6E-06 | 0.57 | 0.033 | |
| 7-Dehydrocholesterol-4 Desmosterol-3 | 367.34 | 16.08 | 1.5E-04 | 4.0E-05 | 7.3E-05 | 2.7E-06 | 0.50 | 0.036 | |
| Sphinganine 1-phosphate | 382.28 | 12.97 | 7.1E-05 | 9.5E-06 | 5.0E-05 | 7.4E-06 | 0.70 | 0.037 | |
| Metanephrine | 198.11 | 7.51 | 3.5E-05 | 1.3E-05 | 6.6E-05 | 7.7E-06 | 1.91 | 0.043 | |

Relative levels of metabolites were estimated by normalizing the areas of the identified peaks to those of the internal standards, assessed using CE-TOF-MS and LC-TOF-MS. Day 0, before consumption; day 29, after consumption of yellow pea noodles; *Significant difference between days 0 and 29; P < 0.05 (Welch's t-test); SD, standard deviation; MT, migration time; RT, retention time.

**Table S3.** Concentration of acetic acid in feces of participants.

| Compound name | Participant No. | Concentration (µmol/g) | | Comparative analysis | |
| --- | --- | --- | --- | --- | --- |
|  |  | Day 0 | Day 29 | | Ratio (Day 29/Day 0) |
| Acetic acid | No.2 | 18.80 | 23.05 | | 1.23 |
|  | No.3 | 28.83 | 69.62 | | 2.41 |
|  | No.4 | 48.14 | 24.27 | | 0.50 |
|  | No.5 | 40.95 | 17.77 | | 0.43 |
|  | No.6 | 54.48 | 67.30 | | 1.24 |
|  | No.7 | 38.45 | 42.00 | | 1.09 |
|  | No.8 | 22.05 | 24.37 | | 1.11 |
|  | No.11 | 54.35 | 59.61 | | 1.10 |
| Mean |  | 38.26 | 41.00 | |  |
| SD |  | 13.92 | 21.63 | |  |
| Average Ratio |  |  |  | | 1.07 |
| P-value |  |  |  | | 0.72 |

Acetic acid content in feces was quantified using colorimetric methods. Day 0, before consumption; day 29, after consumption of yellow pea noodles; data were compared between days 0 and 29 using paired t-test; SD, standard deviation.

**Table S4.** Relative area of propionic acid in feces of participants.

| HMT DB | *m/z* | MT/RT | Participant No. | Relative area | | Comparative analysis |
| --- | --- | --- | --- | --- | --- | --- |
| Compound name |  |  |  | Day 0 | Day 29 | Ratio (Day 29/Day 0) |
| Propionic acid | 73.03 | 9.67 | No.2 | 0.02 | 0.02 | 0.98 |
|  |  |  | No.3 | 0.02 | 0.03 | 1.51 |
|  |  |  | No.4 | 0.02 | 0.02 | 0.74 |
|  |  |  | No.5 | 0.03 | 0.01 | 0.40 |
|  |  |  | No.6 | 0.03 | 0.04 | 1.44 |
|  |  |  | No.7 | 0.02 | 0.02 | 0.81 |
|  |  |  | No.8 | 0.01 | 0.01 | 0.68 |
|  |  |  | No.11 | 0.04 | 0.03 | 0.73 |
| Mean |  |  |  | 0.02 | 0.02 |  |
| SD |  |  |  | 0.01 | 0.01 |  |
| Average Ratio |  |  |  |  |  | 0.89 |
| P-value |  |  |  |  |  | 0.62 |

Relative area of propionic acid was estimated using CE-TOF-MS and LC-TOF-MS. Day 0, before consumption; day 29, after consumption of yellow pea noodles; data were compared between days 0 and 29 using Welch's t-test; SD, standard deviation; MT, migration time; RT, retention time.

**Table S5.** Relative area of butyric acid in feces of participants.

| HMT DB | *m/z* | MT/RT | Participant No. | Relative area | | Comparative analysis | |
| --- | --- | --- | --- | --- | --- | --- | --- |
| Compound name |  |  |  | Day 0 | Day 29 | Ratio (Day 29/Day 0) |  |
| Butyric acid Isobutyric acid | 87.04 | 8.82 | No.2 | 0.01 | 0.01 | 0.78 |  |
|  |  |  | No.3 | 0.02 | 0.06 | 2.86 |  |
|  |  |  | No.4 | 0.02 | 0.04 | 1.95 |  |
|  |  |  | No.5 | 0.03 | 0.01 | 0.41 |  |
|  |  |  | No.6 | 0.05 | 0.09 | 1.70 |  |
|  |  |  | No.7 | 0.04 | 0.03 | 0.69 |  |
|  |  |  | No.8 | 0.02 | 0.01 | 0.67 |  |
|  |  |  | No.11 | 0.09 | 0.04 | 0.51 |  |
| Mean |  |  |  | 0.04 | 0.04 |  |  |
| SD |  |  |  | 0.02 | 0.03 |  |  |
| Average Ratio |  |  |  |  |  | 1.04 |  |
| P-value |  |  |  |  |  | 0.90 |  |

Relative area of butyric acid was estimated using CE-TOF-MS and LC-TOF-MS. Day 0, before consumption; day 29, after consumption of yellow pea noodles; data were compared between days 0 and 29 using Welch's t-test; SD, standard deviation; MT, migration time; RT, retention time.
